# Laron syndrome presenting with severe low bone density for age in a 28-year-old woman: a case report

**DOI:** 10.1097/MS9.0000000000005570

**Published:** 2026-09-10

**Authors:** Manoj Kumar Chaulagain, Devansh Joshi, Rachana Acharya, Rajendra Tamrakar

**Affiliations:** a Kathmandu University School of Medical Sciences, Dhulikhel, Nepal; b Department of Internal Medicine, Dhulikhel Hospital, Kathmandu University Hospital, Dhulikhel, Nepal

**Keywords:** case report, insulin-like growth factor-1, Laron syndrome, osteoporosis

## Abstract

**Introduction:**

Laron syndrome (LS) is a rare, autosomal recessive disorder of growth hormone insensitivity, characterized by proportional short stature and low concentrations of insulin-like growth factor-1 (IGF-1) despite normal or increased levels of growth hormone.

**Case presentation:**

A 28-year-old woman presented with proportional short stature and a markedly low body weight, with a BMI of 14.5 kg/m^2^. Physical examination revealed a characteristic phenotype, including a prominent forehead, saddle-shaped nose and blue-tinted sclera. Investigations revealed low serum IGF-1 levels with elevated levels of growth hormone. Severe vitamin D deficiency and secondary hyperparathyroidism were observed. Differentials of malnutrition and clinical hypothyroidism were systematically ruled out. Management was focused on closed fracture reduction, vitamin D and calcium supplementation, and physical rehabilitation of the patient.

**Clinical discussion:**

Based on the phenotypic presentation and endocrine profile of the patient, a clinical diagnosis of LS was formulated. Unlike common LS presentations, this patient was markedly underweight, suggesting ethnic and nutritional variations. The comorbid vitamin D deficiency likely acted as a disease modifier, exacerbating the skeletal complications in an already compromised skeletal system.

**Conclusion:**

This case underscores the importance of strong clinical acumen in diagnosing rare growth disorders, especially in resource-limited settings where confirmatory genetic testing is not readily available. It also highlights how secondary bone disturbances can exacerbate the presentation, leading to significant functional impairment.

## Introduction

Laron syndrome (LS) is a rare, autosomal recessive condition characterized by inactivating mutations in the growth hormone (GH) receptor, resulting in low concentrations of insulin-like growth factor-1 (IGF-1) despite normal or increased levels of GH[1]. LS was first described in 1966 in Israel among consanguineous Jewish families when children presented with dwarfism but had high GH levels[2]. About 500 individuals are estimated to be living with LS worldwide[3], with large cohorts of documented cases in Israel and Ecuador^[^4,5^]^. Outside of these regions, LS is extremely uncommon, with a few cases reported in India, Sri Lanka, Syria and Iran.^[^6–8^]^

In young children, LS presents with facial features like a flat nasal bridge, protruding forehead and dental deformities. The patients grow to have a markedly reduced head circumference[9], and a proportionally short stature, with adult height −4 to −8 SDs from the median if left untreated[10].

HIGHLIGHTS

- Laron syndrome (LS) can be diagnosed clinically by proportional short stature and an endocrine profile of high growth hormone/low insulin-like growth factor-1.
- The patient’s presentation was atypical due to being markedly underweight (BMI 14.5 kg/m^2^), which contrasts with the common finding of obesity in LS cohorts.
- The case is complicated by severe skeletal issues, including multiple fragility fractures and profound osteoporosis confirmed by extremely low dual-energy X-ray absorptiometry Z-scores and bone mineral apparent density.
- Coexisting severe vitamin D deficiency and secondary hyperparathyroidism likely acted as a disease modifier, greatly worsening the patient’s existing skeletal compromise.
- The case highlights the importance of strong clinical acumen and recognizing phenotypic variability in diagnosing rare growth disorders, especially in resource-limited settings.

Skeletal involvement is a significant aspect of the disease process, as IGF-1 plays an essential role in osteoblast differentiation and the attainment of peak bone mass[11]. While standard dual-energy X-ray absorptiometry (DEXA) often demonstrates decreased areal bone mineral density (BMD) secondary to extreme short stature, literature suggests that volumetric BMD and bone mineral apparent density (BMAD) are typically paradoxically preserved. Therefore, baseline fracture risk is not inherently elevated in well-nourished patients with uncomplicated LS^[^12,13^]^.

We present a case that, to the best of our knowledge, is the first documented Nepali case of LS presenting with severe osteoporosis and multiple fragility fractures. This severe skeletal compromise is multifactorial, resulting from the compounding effects of untreated LS, severe vitamin D deficiency, and secondary hyperparathyroidism. This case expands the recognized skeletal phenotypes of LS, and illustrates how secondary metabolic bone disorders may complicate cases of untreated LS. Furthermore, this report aims to highlight the diagnostic challenges inherent to resource-limited settings. In regions where confirmatory genetic testing is unavailable, establishing a clinical diagnosis based heavily on classical phenotypic presentation and specific endocrine profiles remains a crucial clinical skill. This case has been reported in line with the CARE guidelines[14].

## Case presentation

A 28-year-old female presented with complaints of short stature and progressive skeletal deformities. According to the patient and her family, she was always noticeably shorter than her peers since early childhood. There was no history of consanguinity or similar conditions in her family, and no chronic illness during her childhood. She had no history suggestive of chronic malabsorption or eating restrictions, and the evaluation did not support primary malnutrition.

The patient reported a 12-year history of multiple skeletal fractures. Her first fracture occurred at age 16. Over the next 12 years, she sustained a total of four fractures at sites including the distal radius bilaterally, the right neck of femur, and the left tibia, primarily resulting from low-impact trauma mechanisms such as minor falls from standing height. She consistently experienced delayed healing times, typically taking 12 to 16 weeks to recover, with physical deformities developing at the fracture sites.

On examination, the patient had a proportionate short stature (upper-to-lower segment ratio: 1.04), with a height of 105 cm (mid-parental height: 156 cm) and a weight of 16 kg (BMI = 14.5 kg/m^2^). Her arm span and head circumference were measured to be 110 and 49 cm, respectively. She also presented with distinct craniofacial features, including a depressed nasal ridge, frontal bossing, an underdeveloped mandible and a history of delayed dentition. Inspection of the eyes revealed blue-tinted sclera (Fig. 1). Peripheral lean muscle wasting was observed. Limb examination revealed deformities and abnormal curvatures, especially in the distal leg and distal humerus. Pubertal development was appropriate, with menarche at 13 years, Tanner stage IV sexual maturation, and regular menstrual function.

**Figure 1.**
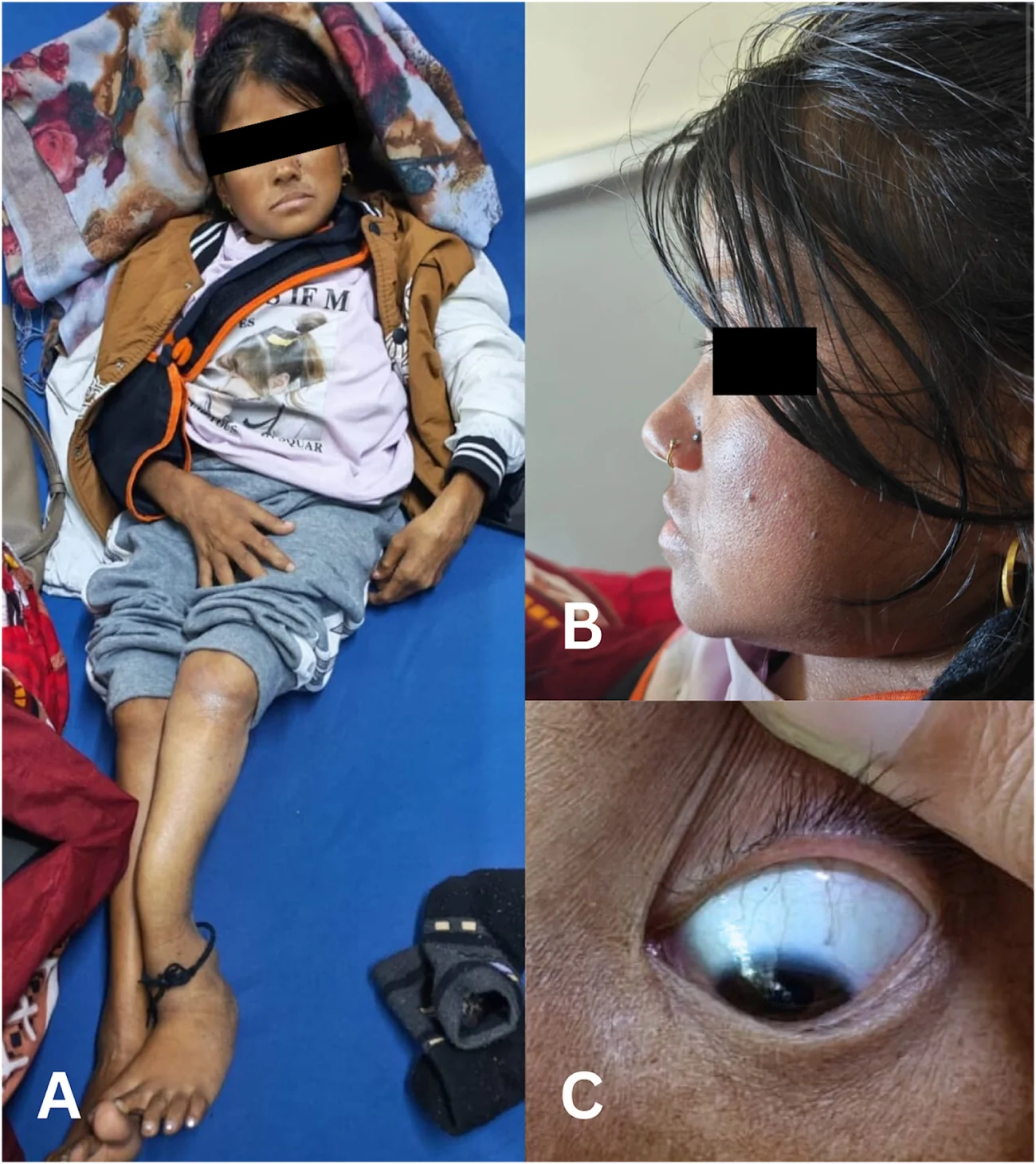
Clinical presentation of the 28-year-old female patient with suspected Laron syndrome. (A) Full-body photograph demonstrating severe proportional short stature. (B) Lateral profile highlighting characteristic craniofacial features, including frontal bossing and a saddle-shaped nose. (C) Close-up view of the patient’s eye revealing a distinct blue-tinted sclera.

Plain radiographs demonstrated severe osteopenia with multiple healed fractures. A complete fracture was present at the distal one-third of the shaft of the humerus (Fig. 2). There was an abnormal curvature in the thoracic spine suggesting scoliosis.

**Figure 2.**
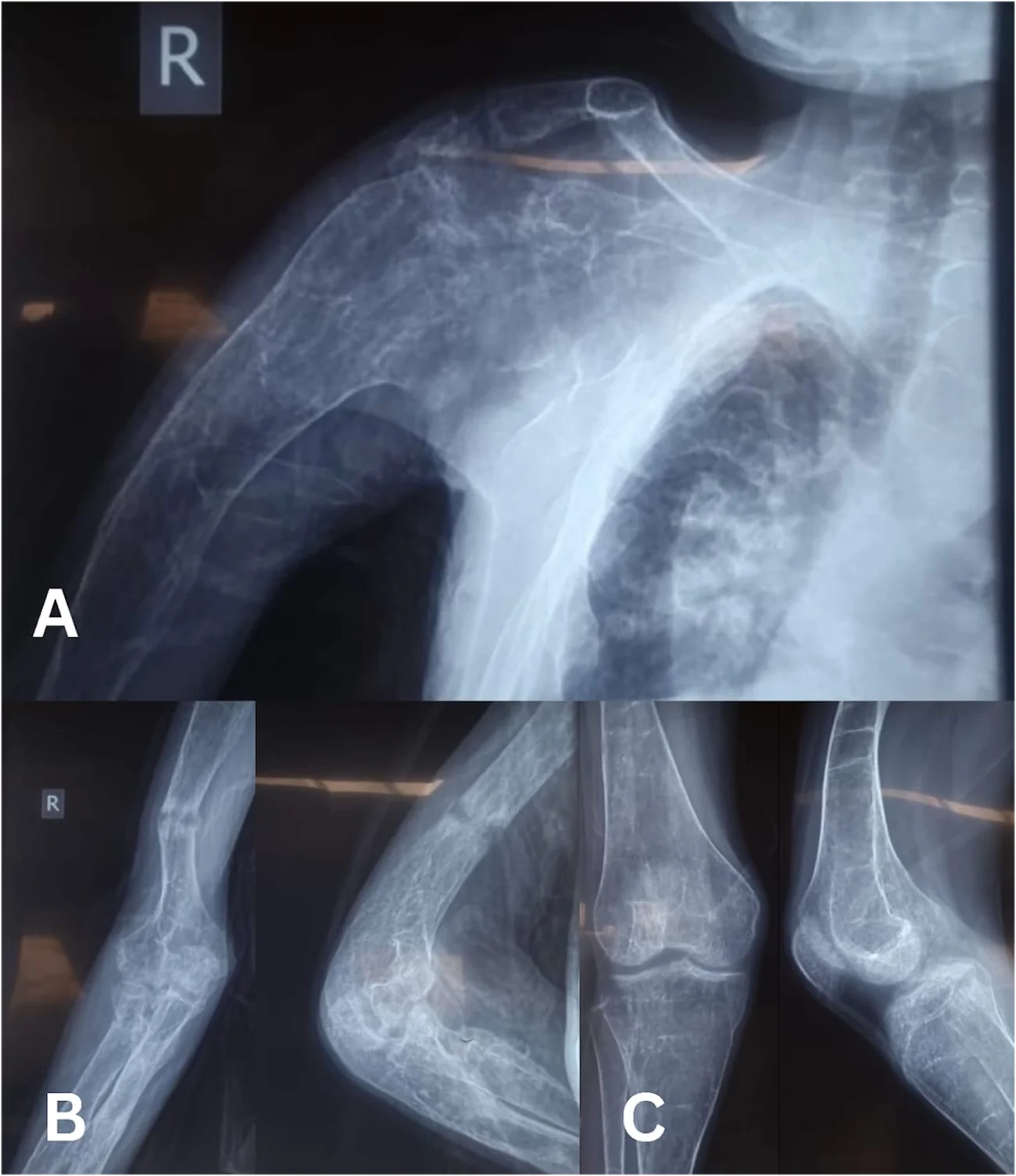
Radiographic findings of the patient’s skeletal system demonstrating severe metabolic bone complications. (A, C) Plain radiographs showing severe generalized osteopenia alongside evidence of multiple healed fractures. (B) Radiograph detailing an active, complete fracture involving the distal one-third of the humeral shaft.

Suspecting a growth disorder, endocrine evaluations were performed. Serum GH levels were found to be elevated, IGF binding protein-3 (IGFBP-3) levels were slightly decreased, while IGF-1 concentrations were markedly reduced, which was consistent with LS (Table 1). Dual X-ray absorptiometry (DEXA) further confirmed severe generalized osteoporosis, with markedly reduced bone mineral density at both her lumbar spine and hips, suggesting a high risk of fractures (Table 2).

**Table 1:** Biochemical findings.

Parameter	Value	Reference range	Interpretation
Growth hormone	15 ng/mL	0.126–9.88 ng/mL	Elevated
IGF-1	46.46 ng/mL	84–289 ng/mL	Low
IGF binding protein-3	4 ug/mL	3.5–7.6 ug/mL	Low-normal
Vitamin D (25-OH)	7.82 ng/mL	10–100 ng/mL	Severe deficiency
Parathyroid hormone	286 pg/mL	4.6–58.1 pg/mL	Markedly elevated
Calcium	8.4 mg/dL	8.4–10.2 mg/dL	Low-normal
Phosphorus	3.3 mg/dL	2.5–4.5 mg/dL	Normal
24-Hour urine calcium	94.35 mg/day	100–300 mg/day	Low
Alkaline phosphatase	1026 IU/L	<105 IU/L (for Adult Females)	Markedly elevated
Sodium	134 mmol/L	135–145 mmol/L	Mildly low
Potassium	3.3 mmol/L	3.5–5.0 mmol/L	Mildly low
Magnesium	2.6 mg/dL	1.7–2.4 mg/dL	Mildly elevated
Total leukocyte count	12 000/mm^3^	4000–10 000/mm^3^	Elevated
Neutrophils	82%	40–70%	Neutrophilia
Hemoglobin	11.5 g/dL	12–16 g/dL	Mildly decreased
CRP	<5 mg/L	<5 mg/L	Normal
AST	19.0 IU/L	5–40 IU/L	Normal
ALT	20 IU/L	5–40 IU/L	Normal
Serum albumin	4.4 g/dL	3.5–5 g/dL	Normal
Creatinine	1.0 mg/dL	0.4–1.1 mg/dL	Normal
Urine protein	Not detected	Not detectable	Normal
TSH	7.87 mIU/L	0.3–3.6 mIU/L	Mildly elevated
Free T4	0.96 ng/dL	0.8–1.7 ng/dL	Normal
Free T3	2.87 pg/ml	2.2–4.2 pg/ml	Normal
Prolactin	22.2 ng/mL	6.20–23.40 ng/mL (Reproductive age females)	Normal
Estradiol	22.3 pg/mL	21–649 pg/mL (Normal Menstruating Females)	Low-normal
Vitamin B12	1390 pg/mL	107–653 pg/mL	Elevated

IGF-1, insulin-like growth factor-1; 25-OH, 25-hydroxy; CRP, C-reactive protein; AST, aspartate aminotransferase; ALT, alanine aminotransferase; TSH, thyroid stimulating hormone.

**Table 2:** Dual-energy X-ray absorptiometry (DEXA) scan results.

Site	BMD (g/cm^2^)	Z-score	BMAD (g/cm^3^)	Interpretation
Lumbar spine (L1–L4)	0.285	−6.9	0.052^a^	Severe osteoporosis
Left femoral neck	0.236	−5.5	N/A^b^	Severe osteoporosis
Left total hip	0.235	−5.8	N/A^b^	Severe osteoporosis

^a^ BMAD was calculated using the formula BMC/Area^1.5^ to minimize the confounding effect of extreme short stature.^11^ This calculated value is significantly lower than the mean lumbar spine BMAD reported by Benbassat et al. for both adult patients with Laron Syndrome (0.201 ± 0.02 g/cm^3^), and healthy controls (0.178 ± 0.03 g/cm^3^), indicating severe true volumetric bone loss.^12^

^b^ BMAD was not calculated at the hip sites due to the lack of validated formulas and data for comparison applicable to our case.

BMD, bone mineral density; BMAD, bone mineral apparent density; L1–L4: lumbar vertebrae 1 through 4; N/A: not applicable.

Further biochemical investigations revealed severe vitamin D deficiency, low-normal serum calcium levels and elevated parathyroid hormone (PTH) levels, indicating secondary hyperparathyroidism. Phosphate levels were within normal limits, magnesium levels were slightly elevated, and alkaline phosphatase was markedly elevated, suggesting high bone metabolism. Notably, laboratory workup also revealed a markedly elevated vitamin B12 level (1390 pg/ml), which was attributed to the patient’s unprescribed use of vitamin supplements for generalized weakness. Liver function tests, morning cortisol levels and random blood glucose levels were found to be normal, with no reported history of seizures during childhood. Additional tests showed mild primary hypothyroidism with increased TSH levels, low-normal estradiol levels, mild electrolyte disturbances, and reactive neutrophilia with no evidence of systemic inflammation. While mild hypothyroidism was noted, clinical evaluation revealed it was largely subclinical and not the primary cause of her extreme short stature.

Abdominal ultrasonography revealed poor corticomedullary differentiation, with the presence of thin-walled cortical cysts bilaterally. An overdistended urinary bladder containing floating echogenic debris was also noted. Clinically, the patient reported an absence of lower urinary tract symptoms, and the post-void residual volume was measured to be minimal, suggesting the bladder overdistension was likely transient, due to holding urine for the examination

Based on her highly characteristic clinical and endocrine profile, a presumptive diagnosis of LS was strongly suspected, and further options were discussed with the family. A comprehensive treatment plan was implemented, including closed reduction and application of a fiberglass U-slab cast for immobilization of the fracture, supplementation of 60 000 IU weekly vitamin D and 1000 mg daily calcium supplements, along with gentle range-of-motion and progressive weight-bearing rehabilitation measures. Regarding her mild hypothyroidism, levothyroxine was withheld initially to reassess her thyroid axis after correcting her severe nutritional and vitamin deficiencies. The patient demonstrated clinical improvement at her 1-month follow-up visit, with progressive healing of her fracture and improving vitamin D, calcium and PTH levels.

## Discussion

This case illustrates the skeletal and metabolic consequences of long-standing IGF-1 deficiency in LS with comorbid conditions like vitamin D deficiency, in resource-limited settings. In the present case, the combination of proportionate short stature, low IGF-1 levels, moderately decreased IGFBP-3 levels, and the presence of elevated levels of GH supports the diagnosis despite the unavailability of genetic testing^[^1,2^]^.

Decreased areal BMD is frequently reported on DEXA scans of LS patients due to extreme short stature[1]. However, literature demonstrates that well-nourished patients with uncomplicated LS typically possess normal or even increased volumetric BMD and BMAD^[^12,13^]^. Consequently, uncomplicated LS does not inherently confer a markedly elevated fracture risk. However, our patient presented with markedly reduced areal DEXA Z-scores, decreased BMAD (Table 2), severe osteopenia on plain radiographs, and multiple healed and an active humeral fracture, indicating significant fragility. The severe, early-onset osteoporosis and multiple fragility fractures observed in our patient highlight a complex clinical scenario where the primary genetic defect was heavily exacerbated by secondary metabolic and nutritional pathologies

The severe skeletal compromise in this patient may be attributed to the synergistic interaction of multiple metabolic bone derangements. Physiologically, IGF-1 is critical for bone acquisition as it acts directly on osteoblasts to stimulate proliferation, differentiation, and the synthesis of bone matrix[11]. Our patient’s profound IGF-1 deficiency essentially halted adequate bone formation. Simultaneously, her profound vitamin D deficiency led to decreased intestinal calcium absorption, triggering a compensatory rise in PTH[15]. This secondary hyperparathyroidism chronically upregulated osteoclast activity, driving aggressive bone resorption[15]. This simultaneous suppression of bone formation and hyperactivation of bone resorption likely acted as a disease modifier, worsening bone demineralization in a condition already characterized by impaired bone formation.

Furthermore, the relationship between the patient’s severely low body weight and her skeletal fragility must be evaluated. With a weight of only 16 kg and a BMI of 14.5 kg/m^2^, the mechanical loading on her skeleton was drastically reduced. According to the mechanostat theory of bone adaptation, adequate mechanical stress is required to stimulate osteocyte signaling and maintain bone mass[16]. The chronic mechanical unloading resulting from her severely underweight state may have acted as an independent catalyst for her generalized osteoporosis.

Metabolic derangements are well described in LS, with an increase in adiposity and obesity documented among patients in previous studies[17]. The pathophysiology regarding the development of obesity in LS remains unclear but studies have suggested an abnormal metabolism of adipose tissue as a result of GH insensitivity and IGF-1 deficiency and is characterized by abnormally high levels of serum adiponectin levels[18]. In contrast, our patient was markedly underweight with a BMI of 14.5 kg/m^2^. While classic LS cohorts usually present with obesity, recent data from South Asia highlight phenotypic variants presenting without generalized obesity^[^8,19^]^. We hypothesize that this phenotypic variability could be driven by a combination of factors: genetic heterogeneity specific to this regional variant of LS, nutritional and environmental factors of South Asia, or potentially a paradoxically high resting metabolic rate unique to this patient.

Given the clinical triad of recurrent fragility fractures, severe generalized osteoporosis, and blue-tinted sclera, osteogenesis imperfecta (OI) was a critical differential diagnosis considered during evaluation. Blue sclerae are classic hallmarks of primary collagenopathies like OI, caused by the thinning of scleral collagen, which allows the underlying choroidal veins to show through[20]. Interestingly, this feature has also been documented as an occasional feature in LS[21]. A highly suggestive distinction favoring LS in our patient lies within the biochemistry of the growth axis as primary OI exhibits normal GH and IGF-1 dynamics[22]. In contrast, our patient demonstrated the pathognomonic endocrine profile of GH insensitivity, including a profoundly suppressed serum IGF-1 and decreased IGFBP-3 levels alongside paradoxically elevated basal GH. This severe disruption of the GH/IGF-1 axis impairs osteoblast differentiation and prevents the attainment of peak bone mass, creating a metabolic bone environment that mimics the skeletal fragility of primary structural bone disorders[1].

Poor corticomedullary differentiation and bilateral renal cortical cysts in the setting of normal RFT findings are not a classical feature of LS and have not been reported previously. Differential diagnoses such as autosomal dominant polycystic kidney disease were evaluated, but found to be highly unlikely due to the absence of relevant family history, normal renal function tests and a lack of renal parenchymal enlargement. These findings are likely incidental simple cysts, with an unknown clinical significance.

Mild endocrine abnormalities, including subclinical hypothyroidism and slightly decreased estradiol levels, were noted during her evaluation. These are unlikely to be major contributing factors to her severe osteoporosis, as the patient also reported a history of regular menstrual cycles, and her estradiol levels were appropriate for the follicular phase of her menstrual cycle. This indicates an intact hypothalamic-pituitary-ovarian axis that adequately protects against estrogen-deficiency-mediated bone resorption.

The limitation of this case is the inability to perform genetic testing. However, recognizing LS and its severe metabolic complications in resource-limited settings presents a significant challenge. We propose the following practical diagnostic and therapeutic algorithm for clinicians lacking access to genetic testing: (1) Maintain a high index of clinical suspicion for patients presenting with proportionate short stature paired with classical craniofacial features (e.g., frontal bossing, depressed nasal bridge); (2) Prioritize targeted endocrine screening specifically for paradoxically high GH alongside profoundly low IGF-1 levels; (3) Immediately screen for concomitant nutritional and metabolic bone diseases, emphasizing Vitamin D, calcium, and PTH profiling; and (4) Institute early, aggressive nutritional rehabilitation and physical therapy. This pragmatic approach can expedite clinical diagnosis and minimize secondary skeletal deterioration even when genetic confirmation is unavailable.

## Conclusion

This case highlights that the severe skeletal phenotypes observed in some patients with LS are not solely dictated by IGF-1 deficiency, but may sometimes be drastically compounded by secondary metabolic bone disorders. Clinicians must prioritize routine screening for Vitamin D deficiency and secondary hyperparathyroidism, alongside aggressive nutritional and physical rehabilitation. Promptly identifying and addressing these modifiable secondary factors is critical to mitigating severe, early-onset osteoporosis and preventing lifelong structural and functional impairments in patients with untreated LS.

## Data Availability

All relevant data are included within the article. The data supporting the findings of this study are available from the corresponding author upon reasonable request.

## References

[1] Laron Z. Lessons from 50 years of study of laron syndrome. Endocr Pract 2015;21:1395–402. 10.4158/EP15939.RA 26401581

[2] Laron Z Pertzelan A Mannheimer S. Genetic pituitary dwarfism with high serum concentation of growth hormone--a new inborn error of metabolism? Israel J Med Sci 1966;2:152–55. 5916640

[3] Laron Z Werner H. Laron syndrome – a historical perspective. Rev Endocr Metab Disord 2021;22:31–41. 10.1007/s11154-020-09595-0 32964395

[4] Laron Z Kauli R. Fifty seven years of follow-up of the Israeli cohort of Laron Syndrome patients—From discovery to treatment. Growth Horm IGF Res 2016;28:53–56. 10.1016/j.ghir.2015.08.004 26307357

[5] Guevara-aguirre J Bautista C Torres C. Insights from the clinical phenotype of subjects with Laron syndrome in Ecuador. Rev Endocr Metab Disord 2021;22:59–70. 10.1007/s11154-020-09602-4 33047268

[6] Chreitah A Hijazia K Doya LJ. Laron syndrome in three female siblings with the development of subclinical hypothyroidism and dyslipidemia in one case: first report of a Syrian family. Oxf Med Case Reports 2021;2021:omab079. 10.1093/omcr/omab079 PMC8436272 34527252

[7] Bitarafan F Khodaeian M Garrousi F. Reporting a novel growth hormone receptor gene variant in an Iranian consanguineous pedigree with Laron syndrome: a case report. BMC Endocr Disord 2023;23:155. 10.1186/s12902-023-01388-1 PMC10357607 37474955

[8] Phanse-gupte SR Khadilkar VV Khadilkar AV. Clinical features and endocrine profile of Laron syndrome in Indian children. Indian J Endocrinol Metab 2014;18:863–67. 10.4103/2230-8210.140236 PMC4192996 25364685

[9] Laron Z Iluz M Kauli R. Head circumference in untreated and IGF-I treated patients with Laron syndrome: comparison with untreated and hGH-treated children with isolated growth hormone deficiency. Growth Horm IGF Res 2012;22:49–52. 10.1016/j.ghir.2012.02.005 22414926

[10] Laron Z Lilos P Klinger B. Growth curves for Laron syndrome. Arch Dis Child 1993;68:768–70. 10.1136/adc.68.6.768 PMC1029371 8333769

[11] Crane JL Zhao L Frye JS. IGF-1 signaling is essential for differentiation of mesenchymal stem cells for peak bone mass. Bone Res 2013;1:186–94. 10.4248/BR201302007 PMC4472101 26273502

[12] Benbassat CA Eshed V Kamjin M. Are adult patients with Laron syndrome osteopenic? A comparison between dual-energy X-ray absorptiometry and volumetric bone densities. J Clin Endocrinol Metab 2003;88:4586–89. 10.1210/jc.2003-030623 14557426

[13] Bachrach LK Marcus R Ott SM. Bone mineral, histomorphometry, and body composition in adults with growth hormone receptor deficiency. J Bone Miner Res 1998;13:415–21. 10.1359/jbmr.1998.13.3.415 9525342

[14] Riley DS Barber MS Kienle GS. CARE guidelines for case reports: explanation and elaboration document. J Clin Epidemiol 2017;89:218–35. 10.1016/j.jclinepi.2017.04.026 28529185

[15] Cunningham J Locatelli F Rodriguez M. Secondary hyperparathyroidism: pathogenesis, disease progression, and therapeutic options. Clin J Am Soc Nephrol 2011;6:913–21. 10.2215/CJN.06040710 21454719

[16] Iwaniec UT Turner RT. Influence of body weight on bone mass, architecture and turnover. J Endocrinol 2016;230:R115–30. 10.1530/JOE-16-0089 PMC4980254 27352896

[17] Laron Z Ginsberg S Lilos P. Body composition in untreated adult patients with Laron syndrome (primary GH insensitivity). Clin Endocrinol (Oxf) 2006;65:114–17. 10.1111/j.1365-2265.2006.02558.x 16817829

[18] Kanety H Hemi R Ginsberg S. Total and high molecular weight adiponectin are elevated in patients with Laron syndrome despite marked obesity. Eur J Endocrinol 2009;161:837–44. 10.1530/EJE-09-0419 19755405

[19] Prasad D Sharma K Singh T. Laron syndrome (LS) – a rare case report. J Clin Biomed Sci 2025;15:136–38.

[20] Maioli M Gnoli M Boarini M. Genotype–phenotype correlation study in 364 osteogenesis imperfecta Italian patients. Eur J Hum Genet 2019;27:1090–100. 10.1038/s41431-019-0373-x PMC6777444 30886339

[21] Cotta OR Santarpia L Curtò L. Primary growth hormone insensitivity (Laron syndrome) and acquired hypothyroidism: a case report. J Med Case Reports 2011;5:301. 10.1186/1752-1947-5-301 PMC3141726 21745362

[22] Marini JC Bordenick S Heavner G. Evaluation of growth hormone axis and responsiveness to growth stimulation of short children with osteogenesis imperfecta. Am J Med Genet 1993;45:261–64. 10.1002/ajmg.1320450223 8456815

